# FusionCancer: a database of cancer fusion genes derived from RNA-seq data

**DOI:** 10.1186/s13000-015-0310-4

**Published:** 2015-07-28

**Authors:** Yunjin Wang, Nan Wu, Jiaqi Liu, Zhihong Wu, Dong Dong

**Affiliations:** Institute of Molecular Ecology and Evolution, SKLEC & IECR, East China Normal University, Shanghai, China; Department of Orthopaedic Surgery, Peking Union Medical College Hospital, Peking Union Medical College and Chinese Academy of Medical Sciences, Beijing, China; Department of Central Laboratory, Peking Union Medical College Hospital, Peking Union Medical College and Chinese Academy of Medical Sciences, Beijing, China

## Abstract

**Background:**

Fusion genes are chimeric results originated from previous separate genes with aberrant functions. The resulting protein products may lead to abnormal status of expression levels, functions and action sites, which in return may cause the abnormal proliferation of cells and cancer development.

**Results:**

With the emergence of next-generation sequencing technology, RNA-seq has spurred gene fusion discovery in various cancer types. In this work, we compiled 591 recently published RNA-seq datasets in 15 kinds of human cancer, and the gene fusion events were comprehensively identified. Based on the results, a database was developed for gene fusion in cancers (FusionCancer), with the attempt to provide a user-friendly utility for the cancer research community. A flexible query engine has been developed for the acquisition of annotated information of cancer fusion genes, which would help users to determine the chimera events leading to functional changes. FusionCancer can be accessible at the following hyperlink website: http://donglab.ecnu.edu.cn/databases/FusionCancer/

**Conclusion:**

To the best of our knowledge, FusionCancer is the first comprehensive fusion gene database derived only from cancer RNA-seq data.

## Introduction

Fusion gene is a chimera product due to the consolidation of two separate genes, which can occur as a consequence of chromosomal structural changes, such as inversion, deletion, amplification or inter-chromosomal/intra-chromosomal translocation [1]. Gene fusion can bring dramatic expression changes compared to previous separate genes because of the regulatory domain displacement, and the resulting chimeric protein-coding transcript will either lose its original function or work into a scabbed protein with functions descended from both its ancestors [2]. As a consequence of accumulation of hereditary variations, cancer can also be the result of gene fusion, especially the fusions related to kinases or transcription controllers [3]. Considering their prevalence and common characteristics across diverse human cancer types, gene fusions are always regarded as a distinct class of ‘mutations’. For example, the recurrent *EML4-ALK* fusion event in lung cancer have been identified [4], and play important role in tumor metastasis. Previously, the fusion events were detected mainly based on RT-PCR, which is not suitable for massively identify fusion genes in cancers.

Although the occurrence of gene fusion events in solid tumor has long been noted, the importance has been realized due to the emergence of next-generation sequencing technology, such as transcriptome sequencing (RNA-seq) [3]. RNA-seq permits genome-wide novel transcript analysis, and spurred gene fusion discovery from diverse human cancers, including prostate, breast, lung and bladder carcinoma [4–7], etc. Up to date, huge amount of cancer RNA-seq data have been available, which provided us an opportunity to comprehensively identify the fusion genes. Meanwhile, algorithms and pipelines provided great convenience for the gene fusion detection [8–11], and many software have been developed with high sensitivity and specificity [12–15]. One obvious benefit of gene fusion discovery based on RNA-seq data is the potential to detect novel gene fusion events.

Several databases of gene fusion have already been issued, such as chimerDB [16] and HYBRIDdb [17]. They are either manually curated database of published literatures or by mapping EST sequences to the human genome to find cancer-related fusion genes in human, which lead to a lower coverage of gene fusions. In this work, we retrieved recently published RNA-seq data in 15 kinds of human cancer, and comprehensively detected the gene fusion events using four gene fusion detection methods. A user-friendly database, FusionCancer, was developed with the attempt to facilitate the cancer gene fusion researches. FusionCancer is provided with an integrated web-based utility, which made our predicted gene fusion events easily accessible to cancer research community.

### Data Content

**Fig. 1:**
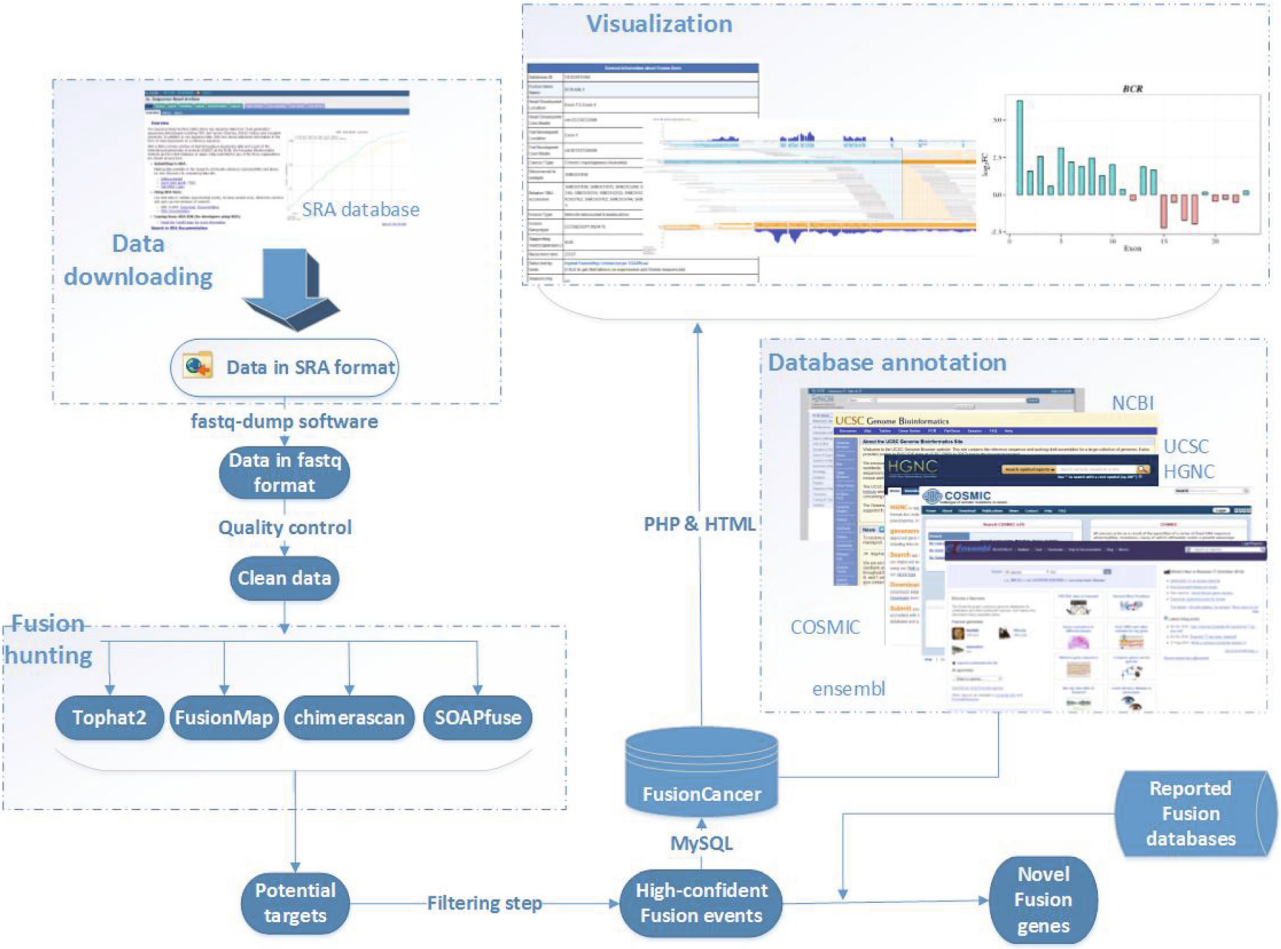
The data acquisition and database construction pipeline

RNA-seq method can allow identification of gene fusions in individual cancer samples and facilitate comprehensive characterization of cellular transcriptome. A huge amount of RNA-seq based cancer transcriptome data are available, which provided valuable resources for us to comprehensively identify gene fusion events in cancers. As shown in Fig. 1, we searched NCBI Sequence Read Archive (SRA, http://www.ncbi.nlm.nih.gov/sra) database [18] for single-end (SE) and pair-end (PE) RNA-seq data in diverse cancer types with the following search terms: ‘cancer’ , ‘carcinoma’ and ‘RNA-seq’. The data included in this work have to meet the following criteria: 1) the length of sequencing reads is larger than 36 bp; 2) the data is cancer-related. Finally, a total of 591 cancer samples, published between 2008 and 2014, were selected for further processing (Table 1).

**Table 1:** List of cancer types and number of RNA-seq dataset included in our work

Cancer types	Number of datasets
Acute Myeloid Leukemia	13
Bladder Cancer	7
Burkitt Lymphoma	52
Cervical Cancer	15
Chronic Myelogenous Leukemia	53
Colon Cancer	18
Hepatocellular carcinoma	22
Intraocular Melanoma	8
Lung Cancer Non-small Cell	18
Lung Cancer	100
Melanoma	27
Neuroblastoma	77
Ovarian Cancer	12
Parathyroid Cancer	7
Prostate Cancer	162
Total	591

The samples included in our database focused on 15 types of cancers. All sequencing raw data were downloaded, and a software named fastq-dump in the SRA Toolkit version 2.3.2 Linux package [19] was obtained from SRA Software page to convert sra format to fastq format with default parameters. We removed the low quality sequencing reads prior to analyzing these data. Two criteria were used in this step: 1) removing reads with adaptors; 2) removing reads with unknown ‘N’ bases. All subsequent analyses to detect fusion genes were based on these filtered sequencing reads.

Fusion gene detection methods have experienced a rapid development due to the emergence of next-generation sequencing technology. In this work, four popular fusion gene detection software (Tophat2 [14], FusionMap [12], SOAPfuse [15], and chimerascan [13] were employed in our pipeline. The former two software can process both single-end and paired-end datasets, while the latter two can only deal with single-end data. We aligned all short reads to the human genome (UCSC hg19), and identified a preliminary set of fusion genes by selecting all the gene-gene pairs. Next, in-build filtering steps were performed, and fusion events were retained if they meet at least one of the following criteria: 1) fusion event formed by two distant genes (with a distance larger than 100000); 2) with a recurrence rate () larger than 0.2; 3) identified by at least two software; 4) containing at least 10 supporting reads. At last, a total of 11,839 gene fusion events were identified based on at least one software, and only 137 fusion genes were identified by all four software. The exon-level expressions of fusion genes and wild-type parts of the fusion genes were calculated by Reads Per Kilobase of transcript per Million mapped reads (RPKM). In addition, COSMIC [20] and chimerDB [16] databases have stored some previously documented known fusion genes in different cancer types. We downloaded 288 fusion genes from COSMIC and chimerDB databases, among which 209 fusion events can be found in our FusionCancer database. So, we implemented these information into FusionCancer.

### Database implementation

The FusionCancer database is implemented with PHP, MySQL on a Red Hat Linux system, and provides several common gateway interface scripts to process user’s input to search the database. A schematic diagram FusionCancer organization is shown in Fig. 1, and FusionCancer can be accessible at the following hyperlink website: http://donglab.ecnu.edu.cn/databases/FusionCancer/

#### Retrieve data

FusionCancer provides two ways to query the database: one is to search fusion genes of interests by keyword, the other is to browse database by cancer types or chromosomes. FusionCancer can be accessed with gene symbols, and return a list of fusion genes, coupled with biological implications, chromosome information and nucleotide sequences. Three kinds of keywords (Gene symbol, Database ID and Gene pairs) can be selected to search fusion gene results. Moreover, users can select fusion genes identified by a specific software. One the other hand, all fusion genes can be viewed through the Browse DB section by choosing a specific cancer type or chromosome.

#### BLAST

To help users perform sequence similarity analysis, BALST was provided in the database. A maximum size of 50 k sequence file with fasta format is required. All fusion sequences at transcription level discovered by four software were used as BLAST database. And users can perform sequence alignment using BLASTn searching form and appropriate parameters listed in the BLAST page.

#### Download

All datasets are available in this section. These datasets contain all predicted fusion genes by four software, accompanied with the annotated information and chimeric transcript sequences.

## Conclusion

RNA-seq is a recently developed way to the transcriptome profiling that uses massively parallel RNA-sequencing technology [21]. The ability of RNA-seq to analyze the whole transcriptome in an unbiased fashion makes it an attractive technology to measure the dysregulation in cancers. Furthermore, it also allows identification of gene fusion in individual cancer samples. With the attempt to provide a more comprehensive cancer fusion gene resource, we compiled recently published cancer RNA-seq data and identified all possible gene fusion events using four popular software. We presented an easily accessible database, offering access to those identified fusion genes. The integration of cancer fusion genes can enhance the role of FusionCancer as an essential resource for cancer fusion gene analysis. To the best of our knowledge, FusionCancer is the first repository centralizing cancer fusion genes identified from RNA-seq datasets. The database not only provides a large resource for cancer researches, but also supplies a platform for tumor specific individual biomarker analysis.

### Future direction

With the development of next-generation sequencing, sequencing costs will drop substantially. More and more cancer sequencing data would be available in the near future, and these data can provide us valuable resource. Moreover, it will be facilitated by the development of improved bioinformatics procedures for the detection of fusion genes from RNA-seq data. Future directions include an incorporation of more cancer fusion genes with higher accuracy.
